# Distinctive Left-Sided Distribution of Adrenergic-Derived Cells in the Adult Mouse Heart

**DOI:** 10.1371/journal.pone.0022811

**Published:** 2011-07-27

**Authors:** Kingsley Osuala, Kathleen Telusma, Saad M. Khan, Shandong Wu, Mubarak Shah, Candice Baker, Sabikha Alam, Ibrahim Abukenda, Aura Fuentes, Hani B. Seifein, Steven N. Ebert

**Affiliations:** 1 Burnett School of Biomedical Sciences, College of Medicine, University of Central Florida, Orlando, Florida, United States of America; 2 School of Electrical Engineering and Computer Science, College of Engineering and Computer Science, University of Central Florida, Orlando, Florida, United States of America; 3 Florida Heart Group, Orlando, Florida, United States of America; University of Chicago, United States of America

## Abstract

Adrenaline and noradrenaline are produced within the heart from neuronal and non-neuronal sources. These adrenergic hormones have profound effects on cardiovascular development and function, yet relatively little information is available about the specific tissue distribution of adrenergic cells within the adult heart. The purpose of the present study was to define the anatomical localization of cells derived from an adrenergic lineage within the adult heart. To accomplish this, we performed genetic fate-mapping experiments where mice with the *cre-recombinase* (*Cre*) gene inserted into the *phenylethanolamine-n-methyltransferase* (*Pnmt*) locus were cross-mated with homozygous *Rosa26 reporter* (*R26R*) mice. Because *Pnmt* serves as a marker gene for adrenergic cells, offspring from these matings express the *β-galactosidase* (*βGAL*) reporter gene in cells of an adrenergic lineage. *βGAL* expression was found throughout the adult mouse heart, but was predominantly (89%) located in the left atrium (LA) and ventricle (LV) (*p*<0.001 compared to RA and RV), where many of these cells appeared to have cardiomyocyte-like morphological and structural characteristics. The staining pattern in the LA was diffuse, but the LV free wall displayed intermittent non-random staining that extended from the apex to the base of the heart, including heavy staining of the anterior papillary muscle along its perimeter. Three-dimensional computer-aided reconstruction of XGAL+ staining revealed distribution throughout the LA and LV, with specific finger-like projections apparent near the mid and apical regions of the LV free wall. These data indicate that adrenergic-derived cells display distinctive left-sided distribution patterns in the adult mouse heart.

## Introduction

Cardiac development is a multifarious process that involves cell specification, determination, patterning, migration, and growth. This is achieved through precise regulation of gene expression within several cell types during embryonic development. The production of signaling molecules designed to coordinate these events during embryonic development is crucial. Among the multitude of signaling molecules are the adrenergic hormones, adrenaline and noradrenaline. Molecular studies of the function of adrenergic hormones during embryonic development have shown that they are vital for cardiac development because mice lacking adrenergic hormones die in utero near midgestation due to apparent heart failure [1].

Adrenergic cells are distributed throughout the developing heart, including regions associated with pacemaking and conduction tissue [2], [3]. In mice, cardiac adrenergic cells appear as early as embryonic day 8.5 (E8.5) [4]. These cells are responsible for the production and secretion of adrenaline and noradrenaline [2], [3], [5]. Immunohistochemical staining experiments for adrenergic biosynthetic enzymes, including tyrosine hydroxylase (Th), dopamine β-hydroxylase (Dbh), and phenylethanolamine n-methyltransferase (Pnmt), indicated transient expression of an adrenergic cellular phenotype in different regions of the heart as development proceeds [3], [6].

Most of the anatomical work with adrenergic cells in the heart has focused on adrenal chromaffin-like cells that are commonly found in “glomus-like clusters” in and around cardiac ganglia from a wide variety of species [7]–[13]. More recent work suggests that adrenergic cells are also found in a subset cardiomyocytes [4], [5], [14]. Indeed, Pnmt enzymatic activity and adrenaline are found in both cardiac atria and ventricles from rats [15]–[19], mice [18], and humans [20]–[22]. Further, Pnmt activity increases dramatically following chemical denervation with 6-hydroxydopamine (6-OHDA), indicating that it is likely expressed in non-neuronal tissue within the heart [23], [24]. The precise anatomical localization of Pnmt-expressing cells within the cardiac atria and ventricles has not been described previously.

In the present study, we explore the distribution of adrenergic-derived cells within the adult mouse heart. To accomplish this objective, we used the previously established *Pnmt-Cre* mouse model, in which the *Cre-recombinase* gene was knocked-in to the *Pnmt* gene locus. When crossing *Pnmt-Cre* mice with *ROSA 26* reporter (*R26R*) mice [25], the *β-galactosidase* (*βGAL*) reporter gene was exclusively activated in adrenergic (*Pnmt*-expressing) cells [4], [26]. It is important to note that cells marked by *βGAL* expression in this model reflect distribution of cells that are derived from an adrenergic lineage even if they no longer actively express *Pnmt* and the other adrenergic enzymes [5], [14]. We utilized standard histological staining techniques combined with computer-aided three-dimensional reconstruction to provide a detailed anatomical characterization of the fate of adrenergic cells in the adult mouse heart.

## Methods

### Materials

All chemicals and reagents were obtained from Sigma-Aldrich (St. Louis, MO) except where noted otherwise.

### Mice

The *Pnmt^+/Cre^* and *ROSA26^+/βgal^* (*R26R*) mice have been described previously [4], [25]. The *Pnmt^+/Cre^* mice were derived after several backcrosses (4–6) with wild-type *129/SvJ* mice (stock # 0006910;Jackson Laboratories, Bar Harbor, ME), and the *R26R* line was backcrossed with wild-type *C57Bl/6* mice (stock #000664; Jackson Laboratories, Bar Harbor, ME) for a similar number of generations (4–6). Each strain was then independently maintained in homozygous condition (*Pnmt^Cre/Cre^* and *ROSA26^βgal/βgal^*, respectively) in the Transgenic Animal Facility at the University of Central Florida where they were housed on a 12∶12 h light∶dark cycle and provided with food and water ad libitum. For all experiments described here, *Pnmt^Cre/Cre^* and *ROSA26^βgal/βgal^* mice were mated, and the resulting heterozygous offspring were studied. All animal experiments were conducted in accordance with the University of Central Florida Animal Care and Use Committee, consistent with regulations for vertebrate animal research outlined by the National Institutes of Health (NIH). The protocol (#08-32) was approved by the University of Central Florida Animal Care and Use Committee on August 18, 2010. This approval has been renewed annually since the initial approval date of August 24, 2008. The title of the protocol is “Molecular imaging of novel cardiomyocyte stem cells”, and it is active through August 17, 2011.

### Histological Preparation and Staining

Adult (8–10 weeks of age) *Pnmt^+/Cre^ ROSA26^+/βgal^* mice were anesthetized with 2.5% isolflurane and sacrificed by decapitation while under full anesthesia. The heart was rapidly removed and perfused retrogradely via a cannula positioned in the aorta. After flushing with phosphate-buffered saline (PBS), the heart was fixed by gentle perfusion with 20 mls of 2% paraformaldehyde in PBS followed by immersion in the same solution for an additional 24 h at 4°C. The hearts were then transferred to a solution of 30% sucrose containing 0.02% sodium azide in PBS for at least another 24 h, until ready for cryostat sectioning. The hearts were then embedded in Tissue-Tek® O.C.T. Compound (EM Sciences, Hatfield, PA) for sectioning using a Microm HM 505 N cryostat set to a temperature of −26°C to −28°C. The hearts were cut at 14–20 microns and mounted onto Super-Frost Plus microscope slides (Fisher Scientific, Inc., Pittsburgh, PA). Tissue sections were stained immediately or stored at −20°C for subsequent use.

Tissue sections were stained for β-galactosidase activity using 50 mM 5-Bromo-4-chloro-3-indolyl- β-D-galactopyranoside (XGAL) dissolved in dimethylformamide as described previously [4]. XGAL was then diluted in XGAL staining buffer solution (5 mM potassium ferricyanide, 5 mM potassium ferrocyanide, 1 mM MgCl_2_, and .01% Tween-20 in PBS) at a dilution factor of 1∶39. Slides were rinsed in PBS for 10 mins and incubated in the diluted XGAL solution overnight at 37°C. The following day, the slides were washed in PBS 3 times for 10 mins each. Sections were counterstained with eosin followed by ethanol dehydration and clearing in xylene for 10 mins before adding a coverslip using Permount (Fisher Scientific, Inc., Pittsburgh, PA). Low-magnification digital images of histology sections were obtained using a Leica MZ16A stereomicroscope and Leica DFC 320 camera system. Higher magnification images were acquired using an upright Nikon Eclipse E600 light microscope with attached SPOT RT™ Slider camera (Diagnostics Instruments, Inc., Sterling Heights, MI).

For immunofluorescent staining experiments, the tissue was prepared as above, and stored at −20°C until use. The general staining procedure was performed as previously described [3], with some modifications as indicated in the following text. The slides were then thawed, heat-dried at 37°C for 2 mins prior to permeabilization in 0.1 N HCL at room temperature for 5 mins followed by a single wash with tap water. The sections were then incubated in PBS containing 10% Tween 20 for 6 h followed by two 15 min washes in PBS without Tween-20. Blocking was performed by incubating in PBS containing 5% fetal bovine serum (Hyclone Labs; Logan, UT) for 30 min prior to incubation for 2 h at 37°C in a humidified chamber with a rabbit anti-Pnmt primary antibody (AB110) from Millipore, Inc. (Temecula, CA) at a dilution of 1∶250 in PBS. In some experiments co-staining with a mouse anti-sarcomeric α-actinin (A7811, Sigma-Aldrich; St. Louis, MO) at a dilution of 1∶100 in PBS was also performed. The sections were then washed twice with PBS alone for 15 min each, followed by 30 min incubation with a FITC-conjugated donkey anti-rabbit IgG (Jackson Immunolabs, Bar Harbor, ME) and/or an Alexa Fluor 594-conjugated goat anti-mouse IgG (Invitrogen, Inc.; Carlsbad, CA). The sections were again washed with PBS as above, then dried and cover-slipped with Vectashield mounting medium (Vector Labs, Burlingame, CA) for fluorescent microscopy.

### 2D Digital Image Analysis

Images were analyzed using Adobe Photoshop™ software. For βGAL quantification, the size of the pictures was adjusted to 669300 pixels (690×970 pixels) per image. This adjustment was confirmed with the image histogram option in the Photoshop software. Once all images were set to the equivalent size, we used the Photoshop “magic wand” to select all blue staining from the left, and then the right side of the heart. Histogram analysis of the βGAL staining on the left and the right side of the heart yielded a total number of blue pixels for each side of the heart. The average pixel value for each individual heart was derived from analysis of 5–10 random sections.

### 3D Image Reconstruction

For a series of 2D slices of the stained heart, we applied a color-based clustering technique to segment regions of interest (i.e., XGAL-stained regions) and then reconstructed the corresponding 3D volume. Among all of the 2D slices, a subset of slices of interest can be manually selected according to different experiential goals. The segmentation for each selected slice went through three steps. First, a slice was converted into the L*a*b* color space. Next, the *k-means* algorithm was employed using squared Euclidean distance to cluster the color feature of the slice into several clusters, where each cluster corresponds to a different color range. Note that the number of clusters, K, is experimentally determined for each slice in view of the intensity inhomogeneity among the slices. While K is initialized for each slice by 6, it can be interactively tuned (increasing or decreasing) in order to find the optimal segment that corresponds to the XGAL-stained regions the best. Then, the optimal segment is extracted according to the labeling index of the corresponding cluster. Repeating the above steps to each slice, we obtained respective XGAL-stained regions for all of the slices of interest. Subsequently, a rigid registration (i.e., translation and rotation) are applied to align the segments obtained from different slices. After that, the aligned segments are sequentially stacked for 3D volume reconstruction. Note that in order to simulate the thickness of a slice, the segmented XGAL-stained regions of each slice are repeatedly stacked for 7 times. The stacked 3D data are saved in MetaImage format (.mhd). Finally, we utilized a free tool called ITK-SNAP (http://www.itksnap.org/pmwiki/pmwiki.php) to load the 3D data and construct the 3D shape of the XGAL-stained volume. ITK-SNAP provided 2D views from axial, sagittal, and coronal directions as well as 3D visualization of the volume that can be observed from dynamic viewpoints.

### Statistical Analyses

All data are presented as the Mean ± S.E.M. Statistical significance was evaluated using the Student's T-test, with *p*<0.05 required to reject the null hypothesis.

## Results

**Figure 1:**
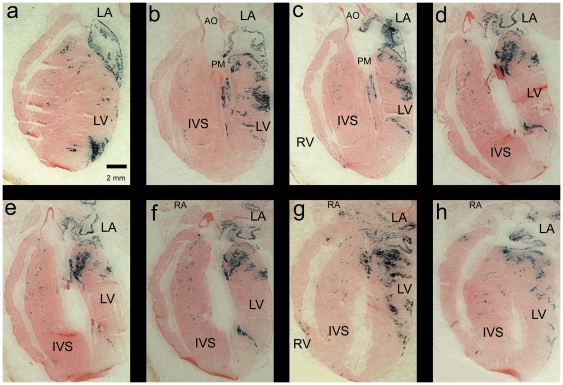
*Pnmt-Cre* activation of β-gal expression in adult mouse heart sections. Representative series of frontal sections (ventral-dorsal orientation) from an adult mouse heart resulting from *Pnmt^Cre/Cre^*, *ROSA^+/+^×Pnmt^+/+^*, *ROSA26^βgal/βgal^* matings to produce offspring that are *Pnmt^+/Cre^*, *ROSA26^+/βgal^*. When the offspring reached adulthood (8–10 weeks), they were sacrificed, and the hearts were fixed and processed for XGAL staining (blue). Similar patterns were seen with hearts isolated at 3–6 months of age (not shown). The tissue was counterstained with eosin (pink) for contrast. Abbreviations: RA, right atrium; LA, left atrium; RV, right ventricle; LV, left ventricle; AO, aorta; PM, papillary muscle; IVS, interventricular septum.

To identify adrenergic-derived cells in the adult mouse heart, we first set up matings between *Pnmt^Cre/Cre^*, *ROSA26^+/+^* and *Pnmt* ^+/+^, *ROSA26^βgal/βgal^* as previously described [4]. The offspring from these matings were allowed to develop normally until adulthood (8–10 weeks), at which time they were sacrificed, and the hearts collected for histological staining analyses. In this model, cells that express *βgal* are indicative of cells that are derived from an adrenergic lineage due to their expression of Cre-recombinase from the *Pnmt* genetic locus. *βgal* expression is readily visualized as blue cells following XGAL histological staining. Examples of this staining are shown in a series of representative adult heart sections in Fig. 1.

### Adrenergic-derived cell distribution in the adult mouse heart

XGAL+ cells were observed in several regions of the myocardium. For example, some of the most strongly positive XGAL staining was consistently observed in the LA; thereby indicating that many of the myocytes in the LA were derived from an adrenergic lineage. The LV was also stained heavily with XGAL in an apparently non-random and non-uniform pattern. In sections from the ventral surface of the heart, we saw an area of strong XGAL+ staining near the apex (Fig. 1a), but as we progressed from ventral towards more dorsal sections, the LV staining pattern appeared to shift first to a more medial location along the longitudinal axis of the heart (Fig. 1, panels b and c), and then continued to shift towards the base of the heart (Fig. 1, panels d–f). In addition, the XGAL staining pattern seemed to generally redistribute from the periphery towards a more interior position when moving from ventral to dorsal locations (Fig. 1, panels b–f). XGAL staining of the papillary muscle (PM) was primarily localized to the superior border, with minimal staining at the right base of the muscle (Fig. 1, panels b and c). XGAL+ staining of the PM was unchanged as the sections were analyzed from ventral to dorsal views. No XGAL+ staining was observed in endothelial cells lining the aorta, pulmonary trunk, or cardiac vessels.

The interventricular septum (IVS) showed intermediate levels of XGAL+ staining with a preferential tendency for localization in the right side of the septum (Fig. 1, panels b–f). This pattern of staining was observed throughout the entire depth of the heart. As seen in Fig. 1, the XGAL+ staining in the body of the IVS was sparse throughout the heart however, we observed a small concentrated cluster of XGAL+ cells along the basal crest of the IVS (Fig. 1, panels d–f). The right ventricular free wall (RVFW) had minimal XGAL+ staining in most sections of the adult mouse heart (Fig. 1, panels a–f). Notably, however, XGAL+ staining in the RVFW was localized to two small areas, one near the apex and another near the base (Fig. 1, panels g and h). These results were consistent in each of the adult mouse hearts analyzed, thus suggesting that while adrenergic-derived cells are primarily localized on the left, there are smaller contingencies of these cells in the IVS and RV.

### Quantification of XGAL+ staining in the adult mouse heart

**Figure 2:**
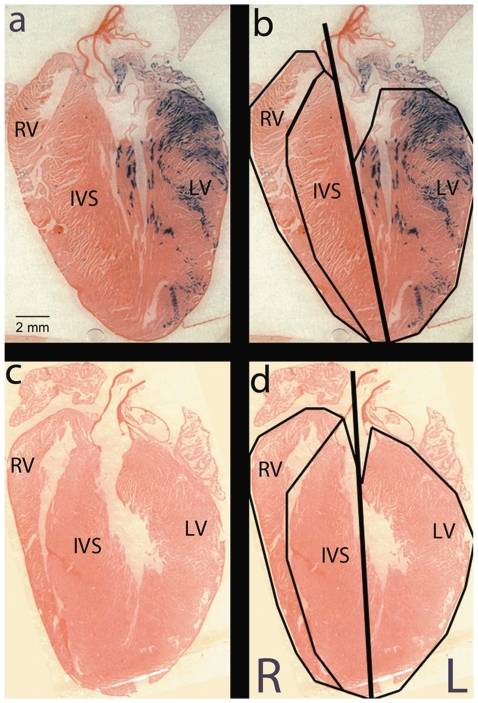
To quantify *Pnmt* expression, *Pnmt* ^+*/Cre*^, *ROSA26* ^+*/βgal*^ adult mouse heart images were partitioned into right and left heart. The line of separation originates from the apex and extends toward the base of the heart and through the aorta (bold line). Additional partitioning isolates the IVS from the RV and LV free walls (panel b and d). Each partition was then analyzed to determine the total pixels containing the blue XGAL+ stain. The left heart shows heavy XGAL+ staining when compared to the right side of the heart. *Pnmt^+/+^*, *ROSA26^βgal/βgal^* control heart shows no XGAL+ staining (panel c and d).

To quantify the XGAL+ staining of the adult mouse heart, we analyzed the total XGAL+ cells in both atria and both free walls of the ventricles. To do this, we first demarcated the major sections of the heart and created a right/left line of separation (Fig. 2). We then quantified blue pixels in images of random sections from each heart. Multiple sections were analyzed from three different hearts, and the results are summarized in Table 1. The combined results show that on average, 89% of XGAL+ cells were found on the left side of the heart. As a negative control, we quantified XGAL+ staining in hearts resulting from *Pnmt^+/+^ROSA26^βgal/βgal^*×*Pnmt^+/+^ROSA26^+/+^* matings (Fig. 2, panels c and d). As expected, no XGAL+ staining was observed in these control hearts. These results demonstrate that XGAL+ staining was predominantly, though not exclusively, present in the atria and ventricular chambers of the left myocardium in the adult mouse.

**Table 1:** Quantification of PNMT/Lac-Z expression in the mouse heart.

	Mean β-Gal positive pixels per five random slices		% β-Gal positive pixels per five random slices	
Heart	Left	Right	Left	Right
R26R-control	0	0	N/A	N/A
R26R-Cre-1	7031±1306	965±412	87.5	12.5
R26R-Cre-2	22806±2568	1827±79	92.2	7.8
R26R-Cre-3	37763±14618	2521±304	87.9	12.1
Mean, R26R-Cre (n = 3)	22533±8873	1771±450	89.2	10.8

*P-value* = .0001.

### 3D computer-aided reconstruction of the Pnmt-expressing cells in the mouse heart

**Figure 3:**
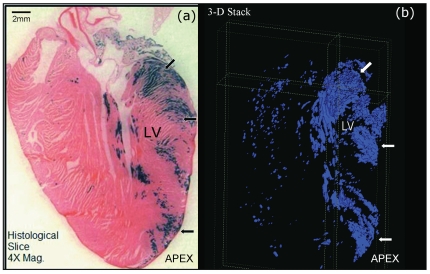
Three-dimensional (3D) reconstruction of the XGAL+ staining in the *Pnmt* ^+*/Cre*^, *ROSA26* ^+*/βgal*^ heart. (a) Representative two-dimensional section of the adult mouse heart were cut at 20 microns. (b) Still-shot of a 3D-rendered image generated from stacked 2D images as described in the methods section. The arrows point to equivalent regions in both panels that roughly correspond to basal, mid, and apical concentrations of XGAL+ cells in these hearts. Please see online supplemental figures 1 and 2 for different perspective views of the 3D image analysis for these data.

To gain a more complete view of adrenergic cell contributions in the adult mouse heart, we examined the 3D distribution of XGAL+ cells in this model utilizing customized computer algorithms to generate three-dimensional representations of the XGAL staining patterns in the adult mouse heart. Representative static views of this analysis are shown in Fig. 3, and a dynamic video sample of these images is provided in Video S1 for LA, and Video S2 for LV. The XGAL staining in the adult mouse heart appears to be largely restricted to the left side, with greater concentration near the base and mid-regions of the LV compared to the apical region. These three-dimensional views confirm the two-dimensional analyses already shown, and provide a more thorough picture of the XGAL staining distribution throughout the adult mouse heart.

### Histochemical and immunofluorescent analyses of adrenergic-derived cells in the adult mouse heart

**Figure 4:**
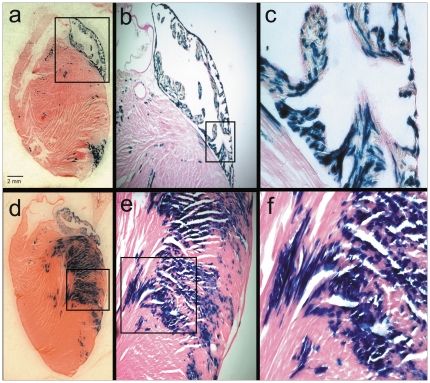
*Pnmt-Cre* expression in cardiomyocytes of the adult mouse heart. XGAL+ staining in the left heart reveals the anatomical location of cells which have historically expressed the *Pnmt* gene. *Pnmt* expression can be seen in the LA and the apical border of the LV ( panel a). *Pnmt* expression is seen distributed throughout the LA and can be seen within the cardiomyocytes under higher magnification (b, c). *Pnmt* expression is also observed in the free wall of the LV and appears to be within the cardiomyocytes, based on anatomical and morphological characteristics (d–f).

**Figure 5:**
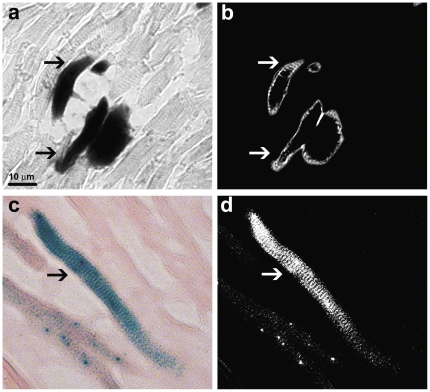
High-magnification phase-contrast and light microscopy images of XGAL-stained cells in the LV free wall from an adult mouse heart. (a) Black and white phase-contrast image of XGAL-stained myocytes (arrows). (b) Color light microscopy image of an XGAL-stained myocyte (arrow). Inverse images of (a) and (b) are shown in panels (c) and (d), respectively. Note the characteristic striated rectangular-like shape of the stained cells.

**Figure 6:**
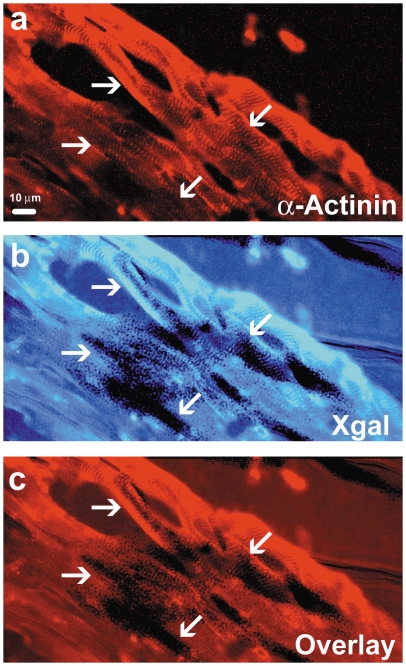
Co-localization of XGAL and sarcomeric α-actinin in LV cells of the adult mouse heart. Immunofluorescent staining for sarcomeric α-actinin in the adult mouse LV shows characteristic striations associated with cardiomyocytes (a, arrows). An adjacent serial section (10 µm) stained with XGAL shows dark (positive) staining, indicating a history of *Pnmt* gene expression (b, arrows). Overlay of the immunofluorescent and XGAL staining show a clear co-localization of cells that have a history of *Pnmt* expression and cells with a cardiomyocyte phenotype (c, arrows).

To obtain a more detailed view of the XGAL+ staining in the adult mouse heart, we captured microphotographs of increased magnification. Representative examples of these results show that most of the cells in the LA were stained with XGAL, especially along the peripheral borders. Similarly, the free wall of the LV was also stained strongly for XGAL (Fig. 4, panels d–f). Anatomical and morphological evaluations of the sections are consistent with a myocyte-specific pattern of staining, though it is also clear that many myocytes remain unstained. Examples of specific myocardial staining were verified with higher magnification phase-contrast and light microscopic images (Fig. 5a and 5c). Inverted image analysis of the stained cells where characteristic sarcomeric striations are clearly evident in the XGAL-stained cells is shown in Fig. 5b and 5d (arrows). In addition, we combined XGAL staining with immunofluorescent labeling of myocardial cells using an anti-sarcomeric α-actinin antibody (Fig. 6). These results clearly show overlapping staining for XGAL and the myocyte marker protein, sarcomeric α-actinin (arrows), thereby confirming that Pnmt-driven XGAL+ staining is found in adult cardiac myocytes in the mouse heart.

**Figure 7:**
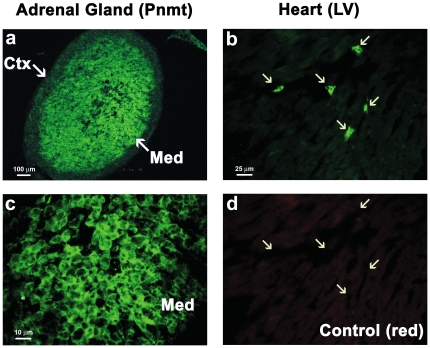
Active *Pnmt* expression in the adult mouse adrenal glands and heart. The adrenal gland and heart of adult *Pnmt^+/Cre^*, *ROSA26^+/βgal^* mice were extracted to evaluate the distribution of active *Pnmt* expression. Cryosections of the mouse adrenal gland were immunofluorescently stained to identify *Pnmt* expression (green). The adrenal medulla (Med) showed bright positive staining as expected, while the cortex (Ctx) of the adrenal gland showed only background fluorescence (a). High magnification images of *Pnmt* immunofluorescent staining in the LV reveals small cells positive for cytoplasmic *Pnmt* expression in the heart (b, arrows) and adrenal medulla (c). Negative control for the LV image shown in panel b illustrates the absence of fluorescent signal in the red spectrum (d).

In this model, XGAL+ staining demarcates active as well as historical expression of Pnmt [4]. To identify cells actively expressing Pnmt in the adult mouse heart, we used an anti-Pnmt antibody to perform immunofluorescent labeling of cardiac tissue sections from adult *Pnmt^+/Cre^*,*ROSA26^+/βgal^* mice. We first tested the specificity of this antibody using adult adrenal gland sections from the same mice. As shown in Fig. 7, cells in the adrenal medulla were brightly labeled with the anti-Pnmt antibody, while the surrounding cortex displayed only background fluorescence (panel a). At higher magnification, the Pnmt immunofluorescent labeling showed characteristic cytoplasmic localization (Fig. 7, panel c), as expected based on earlier immunofluorescent labeling of adrenal chromaffin cells with different anti-Pnmt antibodies used in previous studies [3], [14]. When applied to cardiac tissue, Pnmt immunofluoresent staining identified small triangular-shaped cells that primarily appeared to be located in interstitial spaces (Fig. 7b, arrows). The labeling also appeared to be largely cytoplasmic, and was likely not due to autofluorescence since little or no fluorescent signal was apparent from them upon switching to a red filter (Fig. 7d, arrows). As an additional control, no specific fluorescent staining of either adrenal or cardiac tissue sections was observed in the green spectrum in the absence of the anti-Pnmt primary antibody (not shown). These data indicate that the observed Pnmt immunofluorescent labeling is specific for cells expressing Pnmt.

**Figure 8:**
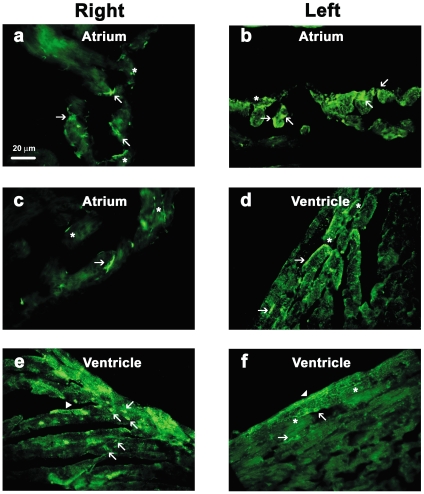
*Pnmt* is expressed in all chambers of the adult mouse heart. Immunofluorescent staining in the adult mouse heart using an anti-*Pmnt* antibody shows active expression in both right and left atria (a–c). The majority of fluorescently labeled cells (green) in the atria have a triangular-shaped appearance (arrows) with neuronal-like extensions (asterisks). In addition, cardiomyocyte-like cells also show positive *Pmnt* staining (arrowheads) which appears to be cytoplasmic while a few cells show staining along their lateral border (d–f). The ventricular myocardium also displayed small triangular-shaped cells which were positive for *Pmnt* expression (e, arrows).

**Figure 9:**
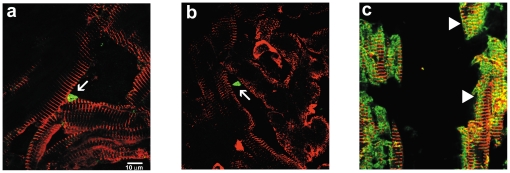
High-magnification confocal co-immunofluorescent staining for Pnmt and sarcomeric α-actinin in adult mouse heart (LV) sections. Pnmt immunofluorescent staining in the adult mouse heart identifies small triangular-shaped cells (green) (a, b arrows) within the interstitial spaces. Cardiomyocyte-specific staining for sarcomeric α-actinin (red) shows identifiable striations in branching cells. Immunofluorescent co-staining with *Pmnt* and sarcomeric α-actinin shows apparent *Pmnt* expression within some cardiomyocytes (c, arrowheads).

To explore this further, we evaluated Pnmt immunofluorescent staining in different regions of the heart as exemplified in Fig. 8. Positive Pnmt immunofluorescent labeling was found within the muscle layers of all four chambers of the heart, where most of them displayed the small triangular-shaped appearance (Fig. 8, arrows). Some of these appeared to have process extensions, possibly suggesting neuronal-like characteristics. In fact, many process-like structures were observed in almost every section (indicated by asterisks, Fig. 8). In a few cases, positive Pnmt immunofluorescent staining appeared to label larger brick-shaped cells in a striated pattern consistent with ventricular myocyte morphological characteristics (Fig. 8e and 8f, arrowheads). We next performed co-immunofluorescent staining of adult mouse heart sections with anti-Pnmt and anti-sarcomeric α-actinin antibodies. When examined at high magnification using confocal laser-scanning fluorescent microscopy, it was clear that the small triangular-shaped Pnmt-positive (green) cells did not co-label with the larger brick-shaped striated myocytes in most cases, but rather were found in close association with the myocytes, typically in the interstitial spaces as shown in Fig. 9 (panels a and b, arrows). A few myocytes, however, appeared to also be positively stained for Pnmt, as indicated by the overlapping Pnmt and sarcomeric α-actinin staining observed (Fig. 9c, arrowheads). These results suggest that Pnmt is actively expressed in cells throughout the heart, and that a small number of these appear to be myocytes, though the vast majority of them appear to be non-myocytes.

## Discussion

In the present study, we showed that cells with a history of expressing the adrenergic biosynthetic enzyme gene, *Pnmt*, were present in the adult mouse heart. This was confirmed by XGAL+ staining in *Pnmt-Cre*×*R26R* mice, where staining was predominantly found in the left atrial and ventricular muscle chambers. While the pattern of staining in the LA was rather diffuse, the distribution of XGAL+ cells in the LV was clearly localized to specific regions. Notably, XGAL staining was heaviest in the basal and mid-sections of the LV, and there was a defined “patch” of strongly stained cells consistently found near midline and apical sections. Staining was not exclusive to these regions, but they represent the major densities of adrenergic-derived cells in the adult heart.

It is important to note that the XGAL-stained cells reflect both active and historical expression of the *Pnmt* gene in the heart because *Pnmt-Cre* acts on the *R26R* locus to activate *βGAL* expression permanently through genetic recombination [4]. Consequently, XGAL+ cells represent the total conglomeration of cells that actively express Pnmt at the time of staining and those that had expressed it at some point earlier in development. Most of the XGAL+ cells in the adult mouse heart appeared to have myocyte-like characteristics based on their distribution, size, brick/rectangular shape, striated patterns, and overlapping staining with a well-established myocyte marker protein, sarcomeric α-actinin.

In contrast, when we exclusively examined active Pnmt-expressing cells using immunofluorescent histochemical staining methods with a specific anti-Pnmt antibody, only a relatively small number of the total Pnmt+ cells in the heart appeared to have myocyte-like characteristics. The vast majority of cardiac cells that were identified by Pnmt immunofluorescent staining were small, triangular-shaped cells located in close association with, but clearly distinct from, myocytes. These Pnmt+ cells were found in all four cardiac chambers where they were most often found in interstitial spaces between bands of myocardial cells. Several examples of process-like staining were observed in Pnmt+ cardiac cells suggesting that some of these may be neurons, though further experiments with additional cell type-specific markers are required to determine if this is true. It is clear, however, that most of the cardiac cells identified as positive for Pnmt immunofluorescent staining do not appear to be myocytes. These results thus suggest that much of the XGAL+ staining likely represents historical expression of the *Pnmt* gene in addition to cells that are actively expressing *Pnmt*.

Previous studies have shown that extracts from the LA display relatively high levels of Pnmt mRNA and enzyme activity compared to the LV [18], [19], [27], and it has been known for many years that Pnmt and adrenaline concentrations are generally higher in the atria than in the ventricles [16], [23], [27]. This appears to be true in human patients as well [20]. Interestingly, there is also evidence indicating that *Pnmt* gene expression is differentially regulated in the atria compared to the ventricles [15]–[18]. Early studies that examined the anatomical localization of putative adrenergic cells (chromaffin-like or small-intensely-fluorescent “SIF” cells), and found them mainly in the epicardium in the glomus or grape-like clusters of extraneuronal cells associated with cardiac ganglia [7]–[13]. Subsequent studies have shown that there is also a significant fraction of Pnmt-expressing cells that are non-ganglionic in both atria [18], and it is well established that chemical destruction of sympathetic nerves with 6-OHDA leads to increases in Pnmt expression and adrenaline production in the heart [23], [24]. Taken together, these previous studies have shown that non-neuronal Pnmt is expressed in the adult heart from a number of mammalian species, including humans. Most of this expression was reported in the atria, especially the LA [18], [19]. Some of this expression may be derived from the small clusters of chromaffin-like cells found in and around cardiac ganglia, but the evidence indicates that there must also be a significant source of these cells that is not associated with ganglia in the heart [17], [20]. The data presented here in combination with previous reports on the origin of adrenergic cells within the developing heart suggest that a significant fraction of these cells are derived from myocardial progenitors [4], [5], [18], [28], while others are likely derived from invading neural crest subpopulations [29], [30].

The present study is the first to demonstrate the cellular distribution of adrenergic cells in adult myocardium. Previous histological examinations focused on chromaffin or SIF cells located in and around ganglia on the epicardial surface of the heart [7]–[10], but no specific cellular staining for Pnmt was reported. Subsequent studies have examined Pnmt mRNA, protein, enzyme activity, and adrenaline formation in the heart [16]–[18], [20], [23], [24], but all of these studies relied on preparation of cardiac extracts. Thus, to the best of our knowledge, this is the first report of detailed anatomical and histological identification of cardiac cells that express Pnmt and those that had a history of expressing Pnmt at some point in their development.

During prenatal development, adrenergic cells are transiently and progressively associated with pacemaking and conduction system centers [3]. In the adult mouse heart, however, we observed only a limited pattern of XGAL+ staining in the right atrium, atrio-ventricular junction, and along the crest and lateral borders of the interventricular septum that are generally consistent with the expected distribution of pacemaking and conduction system tissue. In further contrast to earlier stages of development (i.e., fetal and neonatal) where XGAL+ staining was observed extensively in the muscle cells of all four cardiac chambers [4], the adult staining pattern of Pnmt-driven XGAL staining was clearly much more restricted to myocardium on the left side of the heart.

Interestingly, the left-sided patterning of adrenergic-derived cells in the adult myocardium does not coincide with any known gene expression localization profiles; however, it appears to share anatomical similarity with the ascending segment of the helical ventricular myocardial band (HVMB) described by Torrent-Guasp et al. [31], [32]. Although controversial and not universally accepted [33], [34], the HVMB model describes the directional orientation and intricate looping of myocardium that occurs in order to form two independent ventricles that function as a single unit. The detailed looping described in the HVMB model results in a pair of apical left ventricular loops; ascending and descending, that criss-cross to form a funnel-shaped structure near the tip of the apex. The pattern of XGAL+ staining in the apical region of the adult mouse heart suggests that adrenergic-derived cells could be a component of the ascending fibers of the HVMB model. Additional experiments are required to test the validity of this model.

The presence of adrenergic-derived cells within the adult mouse heart could also suggest involvement of these cells in stress-mediated cardiomyopathies. In particular, the cardiomyopathy known as Tako-Tsubo or “Broken-Heart Syndrome” involves hypokinesis or akinesis of the mid and apical regions of the left ventricle during periods of severe emotional stress [35]–[38]. Patients with Tako-Tsubo typically have symptoms that are similar to myocardial infarction (e.g., chest pain, elevated S-T segment on ECG), but do not display coronary occlusion. Due to the hypokinesis of the mid and apical regions of the left ventricular muscle these regions “balloon” out when viewed by echocardiography, lending Tako-Tsubo another alias, “Apical Ballooning Syndrome” [39], [40]. Although the underlying causes of this syndrome are not well understood, the main hypotheses that have been put forward all involve adrenergic mechanisms [41]–[44]. Our current findings show that adrenergic-derived cells preferentially populate specific portions of the LV free wall, thereby suggesting that these cells could play an important role in the development of certain stress-induced cardiomyopathies such as Tako-Tsubo Syndrome. It is important to note, however, that possible connections to clinical syndromes remain speculative at this point, and that additional experiments are needed to determine the validity of these ideas.

In conclusion, this work demonstrates a distinctive and unexpected left-sided distribution of adrenergic-derived myocardial cells in the adult mouse heart. The specific distribution of these cells in the heart was visualized using 2D and 3D analysis methods to gain a comprehensive map of their anatomical positions. The vast majority of these cells were found on the left side of the heart. While apparently random and uniform in the LA, the pattern of these cells in the free wall of the left ventricular musculature appeared in concentrated bands near the base, mid-section, and apex. Further study is required to determine the potential physiological significance of these unique findings.

## Supporting Information

Video S1 **Dynamic visualization video of 3D** ***Pnmt*** **expression in the adult mouse LA, with respect to the changes in observation viewpoint.**
(WMV)

Video S2 **Dynamic visualization video of 3D** ***Pnmt*** **expression in the adult mouse LV, with respect to the changes in observation viewpoint.**
(WMV)
